# New Eye Lens Dose Limit: Status of Knowledge in Campania Hospitals

**DOI:** 10.3390/ijerph16183450

**Published:** 2019-09-17

**Authors:** Vittoria D’Avino, Leopoldo Angrisani, Giuseppe La Verde, Mariagabriella Pugliese, Adelaide Raulo, Giuseppe Sabatino, Fulvio Coppola

**Affiliations:** 1 National Institute for Nuclear Physics, (INFN), 80126 Naples, Italy; pugliese@na.infn.it (M.P.); raulo@na.infn.it (A.R.); fulvio.coppola@unina.it (F.C.); 2 Department of Computer Science and Electrical Engineering, Federico II University, 80126 Naples, Italy; leopoldo.angrisani@unina.it; 3 Department of Physics “E. Pancini”, Federico II University, 80126 Naples, Italy; glaverde@na.infn.it; 4 Centre for Advanced Metrology and Technological Services (CeSMA), Federico II University, 80126 Naples, Italy; giuseppe.sabatino@unina.it

**Keywords:** cataract, eye lens dose limit, occupational exposure, questionnaire, radiation protection

## Abstract

The International Commission on Radiation Protection (ICRP) in 2011 recommended the lowering of the annual eye lens dose limit from 150 mSv/year to 20 mSv/year in order to reduce the risk of X-ray-induced lens opacity in medical staff. The purpose of this study was to assess the status of knowledge of the new eye lens dose limit and of the radioprotection culture among operators. To this end, a questionnaire was administered to physicians, X-ray technicians, and nurses working in five hospitals of the Campania region, Italy. A total of 64 questionnaires were collected in the hospital departments in which procedures involving ionizing radiation were routinely performed. The data analyzed yielded the following results: 12 operators affirmed to know the new eye lens dose limit, 53 operators routinely wore lead aprons, and 23 operators used lead glasses. Four workers performed eye lens dosimetry through specific dosimeters. A significant lack of knowledge of the reduced eye lens dose limit suggests the need to implement radioprotection-training programs aimed at raising awareness about the importance of health care in the workplace and at reducing the risk of radio-induced effects to the eye lens.

## 1. Introduction

In recent years, the application of X-ray image-guided procedures has rapidly increased in a wide range of clinical areas (i.e., neurosurgery, cardiology, urology, gastroenterology, orthopedics), as they are time-sparing and less invasive for the patient compared with traditional medical procedures [1,2,3]. However, these procedures subject operators to a more intensive and protracted exposure to ionizing radiation. In particular, several studies identified the loss of crystalline lens clarity (i.e., clouding or cataract) as the main risk related to the occupational exposure to radiation [4,5,6,7].

The International Commission on Radiological Protection (ICRP) evaluated the epidemiological evidence for some deterministic effects of radiation exposure, and in the Publication 118, concluded that detectable lens opacity and cataract occurred at dose levels lower than previously considered [8].

For the radiation effects on the eye lens, a threshold of 0.5 Gy for low-Linear Energy Transfer (LET) radiation for acute or protracted exposures was suggested by ICRP as more appropriate than previously assessed (5 Gy for single acute exposures and >8 Gy for protracted exposures). In terms of equivalent dose, the ICRP recommended a reduction for occupational exposure of eye lens from 150 mSv to 20 mSv, averaged over 5 years, with the further restriction of not exceeding 50 mSv in a single year [8,9]. The Health Protection Agency (HPA), the International Atomic Energy Agency (IAEA), and the Council Directive Euratom endorsed the statement of the ICRP and approved the revised dose limit to the eye lens [10,11,12]. Italy had not yet implemented the new dose limit in the national legislation by February 2018 as established by the Council Directive Euratom.

The reduction of the dose limit has a significant impact for different professional figures such as surgeons, medical radiologic technologists (MRTs,) and nurses, involved in several medical activities. Indeed, during X-ray-guided procedures, workers undergo long-time exposure in the operating room.

According to the Italian legislation [13], workers are classified as category A when the risk of exceeding one of the following dose limits is identified: 6 mSv for the annual effective dose, or 45 mSv for the annual equivalent dose to eye lens, or 150 mSv for the annual equivalent dose to the skin and extremities. When the limits of category A are not exceeded, category B is assigned to workers that exceed 1 mSv for the annual effective dose, or 15 mSv for the eye lens, or 50 mSv for the skin and extremities. The dose limits for each exposure class are reported in Table 1.

In order to guarantee the health and safety of workers, dose monitoring is a fundamental tool not only for pathologies connected to the eye lens. To this regard, in 2012, a review study on chronic low-dose radiation exposure highlighted the effects of the cumulative whole-body and head professional lifetime exposure to the brain (cancer and non-cancer) among interventional cardiologists in absence of lead cap protection [14].

The aim of the present study was to assess the status of knowledge regarding the new dose limit for the crystalline and the adoption of X-ray protective equipment and the eye lens dose-monitoring program in several hospitals in the south and east of the Naples district (i.e., Campania region), Italy. The final goal was to collect data in order to improve the planning of radiation protection education and training programs for staff safety in the involved departments. To this end, a questionnaire was designed including items on the knowledge of the new eye lens dose limit, the effective use of individual protection devices (IPD), and eye lens dose monitoring.

Participants were physicians, MRTs, and nurses working at the departments of interventional radiology (IR) including cardiology, hemodynamics, urology, and orthopedics. The questionnaire was administered as a personal interview, and the data were collected anonymously.

## 2. Materials and Methods

### 2.1. Data Collection

Five hospitals located in the south and east of the Naples district were selected to perform the survey. These hospitals offer health care to just over 1 million people. The hospitals were coded S-CA01 to S-CA05. Table 2 lists the hospitals and the departments involved in the study.

Respondents to the questionnaire were exposed workers, that is, physicians, technicians, and nurses. The participants were informed about the aim of the study and that the data would be used anonymously.

### 2.2. Questionnaire Framework

The questionnaire (see Supplementary Materials) consisted of 10 questions concerning different aspects of the radioprotection issue, in particular, knowledge of new Directive 2013/59/EURATOM [11], the use of the protection devices, and dose-monitoring of the eye lens.

The first section of the questionnaire was designed to identify the correlation between specific professionals and the occupational eye lens dose. To this end, the first two questions aimed to collect information on the professional category of the participants (*physician/MRT/nurse*) and exposure classification (*category A/B*). Indeed, the following two questions addressed the knowledge of the new dose limit to the eye lens suggested by the Directive 2013/59/EURATOM [11]. In the case of affirmative response, the source of information (*courses of study/working training/workshop/etc*.) was collected. Then, the participant was asked about the use of individual protection devices (*yes/no*) both for the whole body (*lead apron/thyroid collar*) and for the eye lens (*lead glass/mask/lead cap*), and how often he/she wore them (*always/occasionally/never*). The final questions aimed to assess the performance of crystalline dosimetry (*yes/no*) and, in case of affirmative response, which device (*headband/cap/glass/etc.*) was used to put it and the setting position (*frontal/lateral*) with respect to the radiation beam direction.

In order to identify potential response bias, we checked the truthfulness of participants’ answers with the corresponding department manager and radiation protection officer. In particular, the former confirmed the professional category and use of IPD, while the latter confirmed the exposure category declared by each interviewed worker.

## 3. Results

### 3.1. Collected Responses

**Figure 1:**
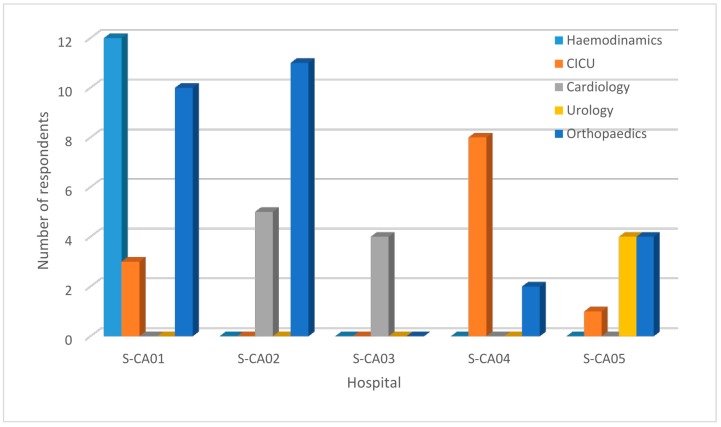
Distribution of respondents across hospitals and departments.

The data were collected between March and April 2019. The wall-targeted population, that is, all workers involved in X-ray-guided procedures, involved 82 operators. Of these, 18 workers refused to participate in the interview. Therefore, 64 questionnaires were completed. The respondents included 21 physicians, 26 MRTs, and 17 nurses. The distribution of the respondents according to their professional categories is shown in Table 3. The number of respondents from each hospital and department is represented in Figure 1.

### 3.2. Workers’ Exposure Classification

The workers’ exposure classification, as they stated, was as follows: 19 physicians and 10 nurses were classified as category A, and 2 physicians together with 26 MRTs and 7 nurses were classified as category B (Table 4).

In the present study, all the interviewed physicians were considered, based on their declarations, as first operator (i.e., the worker closest to the radiation source).

### 3.3. Eye-Dose Monitoring and Use of IPD

**Figure 2:**
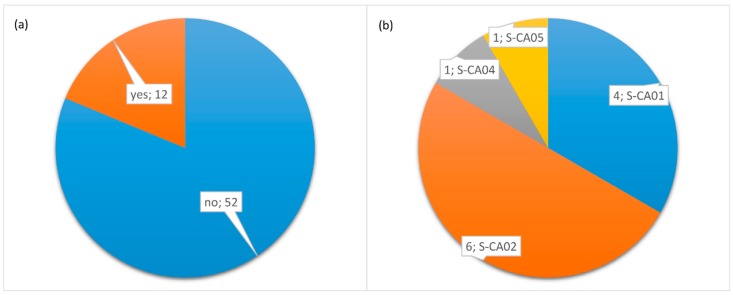
Distribution of workers affirming to know (“yes”) or not to know (“no”) the new eye lens dose limit (**a**), and distribution of yes-respondents across the hospitals (**b**).

Concerning the knowledge of the new eye lens dose limit, 12 respondents declared to be aware of the introduction of a new eye lens annual equivalent dose limit set by the new European Directive. These 12 respondents were distributed as follows: 4 at S-CA01 hospital, 6 at S-CA02 hospital, 1 at S-CA04, and 1 at S-CA05 hospital. All workers affirmed not to know the value of the new dose limit. Figure 2 shows a graphical representation of workers’ responses across the hospitals.

The sources of information reported were mostly personal web research, study courses, workshops, and discussion between colleagues.

From the analyses of the second part of the questionnaire, 53 interviewed workers reported using body radiation protection shields (0.5 mm lead). Among these, 8 workers affirmed to use only body radiation protection shields, 22 workers wore the protective thyroid collar at the same time, 8 workers wore also X-ray-shielding glass, and 15 used both protective thyroid collar and X-ray-shielding glass. Finally, 11 workers (4 physicians, 2 MRTs, and 5 nurses) declared not using any protective device. No worker reported using a lead cap or anti-x mask. All the 53 respondents using at least one type of IPD reported wearing it routinely in their clinical practice.

Concerning dose monitoring, 63 workers stated performing whole-body dosimetry with Thermoluminescent Dosimeter (TLD)-based badges, 15 affirmed wearing also TLD dosimeters as rings or bracelets for dosimetry to the extremities, and 4 operators stated performing dosimetry to eye lens at the same time. Only one worker did not perform any dosimetry.

**Figure 3:**
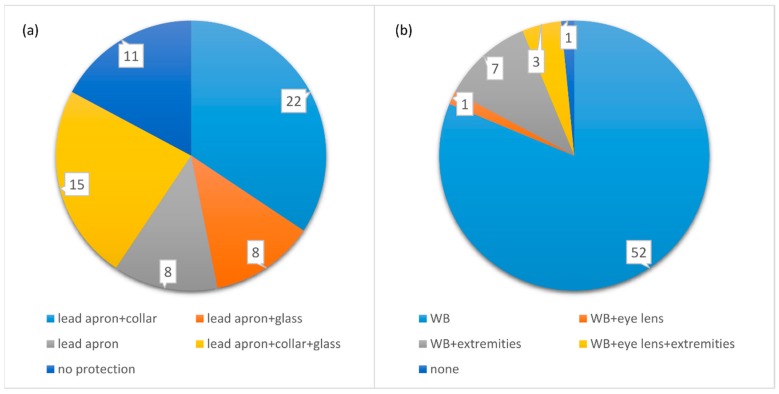
Total number of responses concerning the use of protective shields (**a**) and dosimetry performance (**b**).

The status of use of protective devices and dosimetry is represented in Figure 3.

## 4. Discussion

The results of the present study pointed out that most of the workers do not know that a new limit of dose equivalent to the eye lens will be adopted soon by the Italian legislation in compliance with the indications of the Directive 59/2013. Only two workers declared knowing the recent ICRP statement on the reduction of the annual eye lens dose limit, but they indicated an incorrect value.

To guarantee a faster and more widespread dissemination of the radiation protection indications issued by international organizations and Euratom directives, new tools must be designed. For example, such information can be disseminated not only through periodic radiation protection training programs but also through professional associations, sector-specific newspapers, and lecture notes.

It should be noted that with the new proposed lower limit, the requirements for eye lens dose monitoring and radiation protection measures should be higher. Today, the well-established method to perform eye dose monitoring consists in indirect measurements from a dose badge worn on the trunk (chest level) and inside a lead apron. However, due to the lowering of the eye dose limit, a more accurate and direct dose evaluation is necessary to verify if the cumulative annual dose can potentially exceed the new ICRP dose limit [15,16,17]. In this regard, a previous work on the eye lens radiation exposure of operators in an Interventional Radiology department in Campania, reported that the estimated equivalent dose for the first operator was 54 mSv/year, much higher that the new limit [18]. In the same IR department, a study on the eye lens dose pointed out that the position of the dosimeter affects the exposure dose [19].

Our study evidences that the use of a specific dosimeter for the eye lens is not a common practice in the hospitals involved in the survey. While almost all the respondents (63 out of 64) performed whole-body dosimetry (15 of these also to the extremities), only 4 physicians out of 21 used the eye lens dosimeter for the direct dose measurement. 

In order to reduce the occupational dose, the use of collective and individual protective devices in clinical routine plays a crucial role. In particular, the use of lead glasses for the eye lens can drastically reduce the absorbed dose, since they exhibit a shielding effect up to 60% [15,20,21].

The results of our study showed that despite almost all the respondents (63 out of 64) reporting routinely wearing a lead apron, only 23 stated using shielding glasses. 

Several studies looked into the status of eye dose monitoring, both via questionnaire and/or dose estimation in interventional procedures [3,17,22,23,24,25]. All these studies stated that simple precautions, distribution of roles and functions in the medical team, use of ceiling suspended shields, collective and individual protective devices, and effective dose monitoring programs led to a significant dose reduction. Indeed, the risk of exceeding the occupational eye lens dose limit depends not only on the specific interventional X-ray procedures but also on the specific geometric configuration of C-arms, e.g., the orientation of the X-ray tube and the position of the workers with respect to the X-ray tube [21]. In IR procedures, the workers exposed to a higher dose rate are physicians and nurses who work near the patient. The MRTs received a lower dose than physicians, since they were positioned in a range of 1.7–2.0 m from the X-ray tube.

In order to depict a more complete status, the information obtained in the survey was matched with data previously collected by the local radiation protection officer (RPO). In this way, we found that in some orthopedic departments, the orientation of the C-arm unit and the operators’ position were not adequate to minimize the dose as much as to reasonably achieve the values recommended by the ICRP [12].

However, this study presents some limitations. The sample size was not large enough to generalize the results, and they are representative only of the hospitals included in the survey. In addition, the study is affected by a geographical confinement of the involved hospitals. In order to improve the soundness of the survey, a more extended investigation will be necessary. In particular, we intend to perform the same survey in all the hospitals of the Neapolitan area. However, despite these limitations, to the best of our knowledge this is the first work that investigates the status of knowledge of new eye lens dose limit in the involved hospitals.

## 5. Conclusions

The results of our investigation were concordant with the published literature [17,26,27] and emphasize the need to spread the culture of radioprotection among exposed operators and to revise dose-monitoring in particular for the eye lens. To this end, we stress the importance to design new efficient pathways to spread awareness of the risk associated with medical procedures that use X-rays, and to use personal dosimeters and protective devices. The results highlighted that a policy focusing on radioprotection measures is mandatory to guarantee that recommended occupational dose limits are respected.

In this framework, a closer cooperation between different professional roles (radioprotection experts, physicians, managers, etc.) is fundamental in order to improve the radioprotection strategy in hospital settings. In particular, the availability of adequate protective tools and the adoption of specific cautions during the interventional procedures have to be ensured. Finally, adequate training of physicians, technicians, and nurses, as well as and advice on the correct use of dosimeters and collective/individual protection devices are essential to ensure correct protection and thus the preservation of health in medical working environments as indicated by international regulations.

## Figures and Tables

**Table 1:** Dose limit for each exposure class according to the Italian legislation.

Classification	Effective Dose (mSv/Year)	Equivalent Dose, Skin and Extremities (mSv/Year)	Equivalent Dose, Eye Lens (mSv/Year)
Not exposed worker	≤1	≤50	≤15
Exposed worker category A	>1, ≤6	>50, ≤150	>15, ≤45
Exposed worker category A	>6, ≤20	>150, ≤500	>45, ≤150

**Table 2:** Departments included in the study for each hospital.

Hospital Code	Departments
S-CA01	Cardiovascular Intensive Care Unit (CICU)
	Hemodynamic
	Orthopedics
S-CA02	Cardiology
	Orthopedics
S-CA03	Cardiology
S-CA04	Cardiovascular Intensive Care Unit (CICU)
	Orthopedics
S-CA05	Cardiovascular Intensive Care Unit (CICU)
	Orthopedics
	Urology

**Table 3:** Number (N) of respondents of the five hospitals and their corresponding distribution into the three professional categories: physician, medical radiologic technologist (MRT), and nurse.

Hospital Code	*N*	Professional Category		
		Physician	MRT	Nurse
S-CA01	25	6	12	7
S-CA02	16	7	4	5
S-CA03	4	0	4	0
S-CA04	10	3	3	4
S-CA05	9	5	3	1
Total	64	21	26	17

**Table 4:** Number (N) of respondents in each exposure and professional categories.

Professional Category	Exposure Class		
	A	B	A + B
	N	N	N
physician	19	2	21
MRT	0	26	26
nurse	10	7	17
**Total**	29	35	64

## Supplementary Materials

The following are available online at https://www.mdpi.com/1660-4601/16/18/3450/s1.

## References

[1] Kim K.P., Miller D.L., Berrington de Gonzalez A., Balter S., Kleinerman R.A., Ostroumova E., Simon S.L., Linet M.S. (2012). Occupational radiation doses to operators performing fluoroscopically-guided procedures. Health Phys..

[2] Mahajan A., Samuel S., Saran A.K., Mahajan M.K., Mam M.K. (2015). Occupational radiation exposure from C arm fluoroscopy during common orthopaedic surgical procedures and its prevention. J. Clin. Diagn. Res. JCDR.

[3] Sulieman A., Alzimami K., Habeeballa B., Osman H., Abdelaziz I., Sassi S.A., Sam A.K. (2015). Evaluation of occupational and patient radiation doses in orthopaedic surgery. Appl. Radiat. Isot..

[4] Chodick G., Bekiroglu N., Hauptmann M., Alexander B.H., Freedman D.M., Doody M.M., Cheung L.C., Simon S.L. (2008). Risk of cataract after exposure to low doses of ionizing radiation: A 20-year prospective cohort study among US radiologic technologists. Am. J. Epidemiol..

[5] Junk A.K., Haskal Z., Worgul B.V. (2004). Cataract in interventional radiology - an occupational hazard?. Investig. Ophthalmol. Vis. Sci..

[6] Vano E., Kleiman N.J., Duran A., Rehani M.M., Echeverri D., Cabrera M. (2010). Radiation cataract risk in interventional cardiology personnel. Radiat. Res..

[7] Ciraj-Bjelac O., Rehani M.M., Sim K.H., Liew H.B., Vano E., Kleiman N.J. (2010). Risk for Radiation-Induced Cataract for Staff in Interventional Cardiology: Is There Reason for Concern?. Catheter. Cardiovasc. Interv..

[8] Stewart F.A., Akleyev A.V., Hauer-Jensen M., Hendry J.H., Kleiman N.J., Macvittie T.J., Aleman B.M., Edgar A.B., Mabuchi K., Muirhead C.R. (2012). ICRP publication 118: ICRP statement on tissue reactions and early and late effects of radiation in normal tissues and organs--threshold doses for tissue reactions in a radiation protection context. Ann. ICRP.

[9] (2007). The 2007 Recommendations of the International Commission on Radiological protection. ICRP Publication 103. Ann. ICRP.

[10] Bouffler S., Ainsbury E., Gilvin P., Harrison J. (2012). Radiation-induced cataracts: The Health Protection Agency's response to the ICRP statement on tissue reactions and recommendation on the dose limit for the eye lens. J. Radiol. Prot..

[11] Council Directive 2013/59/Euratom of 5 December 2013 laying down basic safety standards for protection against the dangers arising from exposure to ionising radiation, and repealing Directives 89/618/Euratom, 90/641/Euratom, 96/29/Euratom, 97/43/Euratom and 2003/122/Euratom. https://eur-lex.europa.eu/LexUriServ/LexUriServ.do?uri=OJ:L:2014:013:0001:0073:EN:PDF.

[12] European Commission Faaootun, International Atomic Energy Agency, International Labour Organization, Oecd Nuclear Energy Agency, Pan American Health Organization, United Nations Environment Programme, World Health Organization (2014). Radiation Protection and Safety of Radiation Sources: International Basic Safety Standards.

[13] Johnson D., Johnson R. (2002). Learning Together and Alone: Overview and Meta-analysis. Asia Pac. J. Educ..

[14] Picano E., Vano E., Domenici L., Bottai M., Thierry-Chef I. (2012). Cancer and non-cancer brain and eye effects of chronic low-dose ionizing radiation exposure. BMC Cancer.

[15] Haga Y., Chida K., Kaga Y., Sota M., Meguro T., Zuguchi M. (2017). Occupational eye dose in interventional cardiology procedures. Sci. Rep..

[16] Liu Y.R., Huang C.Y., Hsu C.H., Hsu F.Y. (2017). Dose estimation of eye lens for interventional procedures in diagnosis. Radiat. Phys. Chem..

[17] Carinou E., Ginjaume M., O’Connor U., Kopec R., Sans Merce M. (2014). Status of eye lens radiation dose monitoring in European hospitals. J. Radiol. Prot. Off. J. Soc. Radiol. Prot..

[18] Pugliese M., Amatiello A., Correra M., Stoia V., Cerciello V., La Verde G., Roca V., Loffredo F., Fiore F. (2018). Evaluation of the current status of the eye lens radiation exposure in an Interventional Radiology Department. Med. Lav..

[19] Liverani A., Loffredo F., Fiore F., Correra M., La Verde G., Pugliese M. (2018). Evaluation of the dose dependence on the eye lens from the position of the dosimeter for the operators exposed in interventional radiology. Nuovo Cim. C-Colloq. Commun. Phys..

[20] van Rooijen B.D., de Haan M.W., Das M., Arnoldussen C.W.K.P., de Graaf R., van Zwam W.H., Backes W.H., Jeukens C.R. (2014). Efficacy of Radiation Safety Glasses in Interventional Radiology. Cardiovasc. Interv. Radiol..

[21] Principi S., Farah J., Ferrari P., Carinou E., Clairand I., Ginjaume M. (2016). The influence of operator position, height and body orientation on eye lens dose in interventional radiology and cardiology: Monte Carlo simulations versus realistic clinical measurements. Phys. Medica.

[22] Domienik J., Brodecki M., Rusicka D. (2012). A study of the dose distribution in the region of the eye lens and extremities for staff working in interventional cardiology. Radiat. Meas..

[23] Mesbahi A., Rouhani A. (2008). A study on the radiation dose of the orthopaedic surgeon and staff from a mini C-arm fluoroscopy unit. Radiat. Prot. Dosim..

[24] O’Connor U., Walsh C., Gallagher A., Dowling A., Guiney M., Ryan J.M., McEniff N., O’Reilly G. (2015). Occupational radiation dose to eyes from interventional radiology procedures in light of the new eye lens dose limit from the International Commission on Radiological Protection. Br. J. Radiol..

[25] Vanhavere F., Carinou E., Domienik J., Donadille L., Ginjaume M., Gualdrini G., Koukorava C., Krim S., Nikodemova D., Ruiz-Lopez N. (2011). Measurements of eye lens doses in interventional radiology and cardiology: Final results of the ORAMED project. Radiat. Meas..

[26] Andreassi M.G., Piccaluga E., Guagliumi G., Del Greco M., Gaita F., Picano E. (2016). Occupational Health Risks in Cardiac Catheterization Laboratory Workers. Circ. Cardiovasc. Interv..

[27] Gabrani A., Hoxha A., Simaku A., Gabrani J.C. (2015). Application of the Safety Attitudes Questionnaire (SAQ) in Albanian hospitals: A cross-sectional study. BMJ Open.

