# Supplementary material for: New Eye Lens Dose Limit: Status of Knowledge in Campania Hospitals
Source: Int J Environ Res Public Health. 2019 Sep 17;16(18):3450. doi: 10.3390/ijerph16183450 (PMC6765950; doi:10.3390/ijerph16183450)
Supplement: Supplementary file 1 [file ijerph-16-03450-s001.pdf]

INTERVIEW TO MEDICAL STUFF on the use of RADIOPROTECTION MEASURES

HOSPITAL:

DEPARTMENT:

1) What Professional category do you belong to? *Physician/MRT/Nurse*
2) What is your Classification? *Category A/category B*
3) Do you know the new limit of the equivalent dose to the eye lens proposed by the Directive EURATOM 59/2013? *Yes/No*
4) (in the case of affirmative answer to the previous question) what is the value of the new limit? ---
5) From which source did you learn this information? *courses of study/working training/workshop/etc.*
6) Do you use the individual protection devices (IPD)? *Yes/No*
7) (in the case of affirmative answer to the previous question) what personal protective equipment do you use? l*ead apron/ thyroid collar/ lead glass/mask/lead cap*
8) Do you perform the eye lens dosimetry? *Yes/No*
9) (in the case of affirmative answer to the previous question) which device do you use to fix the dosimeter? *headband/cap/glass/etc.*
10) Which is the setting position of the dosimeter respect to the device? *frontal/lateral*
